# A life course perspective on disability: education-to-work transitions of people with visual impairments in China

**DOI:** 10.3389/fsoc.2025.1627946

**Published:** 2025-11-12

**Authors:** Minjie Chen

**Affiliations:** School of Sociology and Social Policy, University of Nottingham, Nottingham, United Kingdom

**Keywords:** education, life course, transition, people with visual impairment, work

## Abstract

This study adopts a life course perspective to investigate the educational and career trajectories of 26 individuals with visual impairments in China, with a focus on transitions into education and work. Through qualitative semi-structured interviews with participants recruited via snowball sampling from urban and rural areas, thematic analysis identified five patterns of education-to-work transitions, guided by life course principles. Findings reveal delayed educational transitions, high dropout rates (53.88% during compulsory education), and predominant entry into the *tuina* (Chinese medicine massage) industry due to societal stereotypes and structural constraints. Urban participants accessed non-massage skills through family support, enabling diverse careers, while rural participants faced financial pressures to pursue *tuina* for stability, illustrating the principle of linked lives. The study contributes to Chinese disability studies by applying life course theory in a non-Western context, highlighting interactions between timing, sequence, agency, and social forces. Policy implications include expanding inclusive preschool access, mainstream school accommodations, and diverse tertiary programs to broaden career options and challenge vocational stereotypes. Future research should prioritize women with visual impairments to address gender disparities in educational access.

## 1 Introduction

The life course perspective describes age-related developmental stages and the dynamic trajectories of an individual's life (Elder, 1994). Historically, individuals with disabilities in China and globally have encountered significant social barriers due to their impairments, including restrictive public policies, inaccessible environments, and discriminatory social norms, attitudes, and practises. These barriers have often denied them access to education, employment, and decision-making opportunities, resulting in their exclusion from mainstream society (Stone, 2003; Hyde and Shand, 2013).

### 1.1 People with visual impairment in China

According to the Second National Sample Survey of Disabilities in China, conducted in 2006, approximately 82.96 million people, or 6.34% of the national population, live with disabilities, including 12.33 million with visual impairments (The Leading Group for the Second National Sample Survey of People with Disabilities, 2006). Following China's economic reforms in 1978, policies began recognising the equal rights of people with disabilities to societal participation, expecting them to contribute to the nation's competitive, economically driven development alongside their peers without disabilities (Qu, 2020). Influenced by the belief that “blind individuals possess a keen sense of touch and concentration, making them well-suited for massage work” [China Disabled Persons' Federation (hereafter CDPF), 1997, as cited in Tie et al., 2011], the state has promoted the massage industry through policy initiatives to create employment opportunities for individuals with visual impairments. This focus has significantly shaped their educational and career choices, with massage training programmes gradually introduced in schools nationwide (Tie et al., 2011). However, this emphasis may limit career diversity by prioritising massage training over other vocational options (Xiong and Zheng, 2021).

In education, the Compulsory Education Law of 1986 mandates 9 years of compulsory schooling for children starting at age six (or seven in some regions), comprising 6 years of primary education and 3 years of junior high school (The Standing Committee of the National People's Congress, 1986). Special education remains the primary avenue for students with visual impairments, offering both academic instruction and vocational training, such as Chinese medicine massage courses (known as *tuina* 推拿) tailored to their needs (Liu, 2018). Since the mid-1980s, the government has expanded vocational education, particularly massage training, enhancing its accessibility and quality for students with visual impairments (Hua, 2021). However, enrolment in these programmes shows a gender imbalance, with significantly more male than female students (Liao and Chen, 2018). The “Learning in the Regular Classroom” (*suiban jiudu* 随班就读) initiative, introduced in the 1990s, enabled some children with visual impairments, particularly in rural areas, to attend mainstream schools (Ellsworth and Zhang, 2007). Yet, this initiative often provides mere placement in mainstream classrooms without comprehensive, accessible services (Hu, 2022).

The 2006 National Sample Survey reported that 63.19% of children with disabilities were enrolled in either mainstream or special schools during compulsory education, compared to approximately 99% of peers without disabilities. For students with visual impairments, the enrolment rate was notably lower at 31.9% (The Leading Group for the Second National Sample Survey of People with Disabilities, 2006; Hu, 2022). In contrast, the 2021–2025 Special Education Plan aims to achieve a 95% enrolment rate for students with disabilities in compulsory education by 2025 (The Ministry of Education, 2022). For those graduating from special schools, a minority of students with visual impairments take the national college entrance examination (*Gaokao* 高考), which has been available in Braille since 2014 (Chen et al., 2017). In 2025, only 16 candidates with visual impairments registered for the *Gaokao* among 13.35 million total examinees (The Ministry of Education, 2025). Additionally, some junior colleges and universities offer specialised entrance exams for students with visual impairments, though these are limited to subjects such as massage and piano tuning (Ma, 2014).

In employment, while individuals with visual impairments work in various fields, the majority are employed in the massage industry (Liu and Lei, 2017). Working conditions in this sector are often challenging, with long hours (typically 10 h daily), irregular schedules, high labour intensity, and poor living conditions, as many workers reside in massage parlours due to unaffordable housing (Guo, 2017). Another common vocational stereotype involves the creative industries, notably piano tuning (Xiong and Zheng, 2021). Additionally, gender roles impact women with visual impairments, with many young female massage workers viewing marriage as a pivotal career turning point (Xiong and Zheng, 2021).

### 1.2 The life course theory

#### 1.2.1 The life course perspective

The life course is defined as “a sequence of socially defined events and roles that the individual enacts over time” (Giele and Elder, 1998, p. 22), emphasising the interplay between personal experiences and historical and socioeconomic contexts. This perspective examines individuals' life histories, exploring how early experiences shape later life events, such as employment and marriage (Klein and White, 1996). Elder (1994) identified four key factors influencing personal development within the life course: (a) historical and geographic location, which highlights the time and place of an individual's birth and the associated sociocultural contexts; (b) linked lives, emphasising the interdependence of human relationships; (c) human agency, reflecting individuals' ability to make choices and pursue goals; and (d) timing of lives, focusing on variations in the timing of life events and transitions. These factors situate individual and cohort experiences within broader sociocultural conditions, illustrating how early events influence later choices and how individuals respond to social changes (Hutchison, 2011; Heller and Harris, 2011).

Life course trajectories are often shaped by institutional and cultural expectations, with individuals expected to complete transitions such as leaving home, completing education, entering employment, marrying, parenting, and retiring (Shanahan, 2000; Furstenberg et al., 2004). These transitions' sequence and timing create distinct pathways across life stages (Macmillan, 2005). For individuals with disabilities, disability can significantly affect these transitions, potentially impacting subsequent or simultaneous life stages (Tisdall, 2001; McGrath and Yeowart, 2009).

Trajectories and transitions are closely linked, with transitions embedded within broader trajectories, such as education, marriage, or employment, which reflect long-term patterns of stability and change (Elder et al., 2003). Transitions, marked by events like starting school or marrying, alter roles and statuses, often involving family dynamics (Hutchison, 2019). The interaction of trajectories and transitions can create turning points—significant life events that may redirect life trajectories, such as new opportunities or changes in personal beliefs (Rutter, 1996; Li, 1999). These turning points vary in impact across individuals and families (Hutchison, 2011).

#### 1.2.2 Understanding disability through the life course

Traditional disability studies often segment the lives of individuals with disabilities into discrete stages, focusing on specific age groups rather than viewing ageing as a continuous, dynamic process (Janus, 2009; Elder and Giele, 2009). This approach risks oversimplifying human experiences (Settersen, 2003). Similarly, focusing solely on the collective oppression of individuals with disabilities overlooks how institutions and cultures differentially affect them across time and place (Priestley, 2013; Dillaway et al., 2023). Both personal impairments and social barriers shape the life course of individuals with disabilities, and separating these factors limits understanding of their developmental pathways (Erickson and Macmillan, 2018).

The life course perspective, by contrast, captures the complexity of multiple, simultaneous, or sequential role pathways across social institutions (Macmillan and Eliason, 2003). Individuals adopt role-specific behaviours when entering new institutions (e.g., employment or parenthood), sometimes exiting or balancing prior roles (Erickson and Macmillan, 2018). For instance, individuals with visual impairments may navigate roles as students, workers, or caregivers, requiring them to balance multiple responsibilities. This perspective highlights how life trajectories result from the interplay of human agency and social forces, offering a nuanced approach to understanding disability across the lifespan (Elder et al., 2003; Levy et al., 2005; Heller and Harris, 2011; Dillaway et al., 2023).

### 1.3 Overview of the present research

Although recent studies have explored the education and employment of individuals with visual impairments in China, few have done so in the past 5 years, and national data on disability education remain outdated since 2006. The lack of age-disaggregated census data hinders accurate estimation of primary and secondary school enrolment rates for students with visual impairments. Beyond low enrolment rates, there is limited research on their schooling experiences in both special and mainstream settings. While the massage industry dominates employment for this population, little is known about their transitions into this field or alternative career paths.

This study examines the lived experiences of individuals with visual impairments in Northern China, particularly Henan Province. It amplifies their voices—such as those facing barriers to education—to assess the relevance of findings to similar socioeconomic contexts. By adopting a life course perspective, it explores their educational and occupational trajectories, focusing on pathways into the massage industry and other vocational options. In doing so, the research enhances understanding of their schooling and work experiences and contributes to policy discussions on inclusive education, social support, and vocational transitions for individuals with disabilities in China.

## 2 Methods

This study examined the experiences of 26 people with visual impairments aged 25–40 years old with working experience in China. This age range can ensure that participants' lived experiences were all broadly taking place under similar socio-political and cultural contexts (the economic reform era), and following critical pieces of pro-disability legislation and guidance had been put in place. Among participants, three have partial vision and 23 have no vision at the time of the interview, though some had some vision in earlier periods of their lives. Of the 26 participants, 19 were male and 7 were female, primarily from Henan Province in Northern China, representing both rural (*n* = 16) and urban (*n* = 10) backgrounds.

This study involved 26 participants, a sample size considered adequate for rigorous in-depth qualitative inquiry (Guest et al., 2006). Participant recruitment continued until data saturation was reached, defined as the point at which additional interviews yielded no new themes or insights (Fusch and Ness, 2015). Analysis indicated that thematic convergence emerged after approximately 22 interviews, with four further interviews conducted to confirm saturation. The final sample, therefore, provided a sufficient and diverse dataset to comprehensively address the research questions, ensuring both credibility and trustworthiness in line with qualitative research standards.

Data was gathered through interviews conducted in 2022, which took place over the phone due to COVID-19 restrictions in China at that time. Participants were recruited using purposive sampling, with initial contacts being made through referrals (i.e., asking previously recruited participants to share the researcher's contact details with potential peers) (Silver, 2019), as well as snowball sampling to recruit eligible people with visual impairments. The interview questions covered topics such as work experience, school life, family dynamics, access to welfare and benefits, and social barriers and challenges.

All interviews were conducted in Chinese, and transcriptions were completed shortly after each session. Participants were allowed to review their interview transcripts (verbatim) and provide feedback on accuracy, ensuring their narratives were faithfully represented. Data analysis was conducted using these transcripts, and key themes and illustrative narratives were later translated into English. To ensure conceptual equivalence and accuracy in translation, the transcripts were translated by a bilingual researcher and reviewed by a second bilingual expert. Back-translation was employed for select excerpts to verify consistency between the original Chinese and the English translation. Narrative data analysis (Riessman, 2008) was used to analyse how participants transition to education and work.

This study obtained ethical approval from the Research Ethics Committee at the University of Nottingham's School of Sociology and Social Policy (Reference: 2122-14-PGR). Considerations included how to collect data and ensure safety during COVID-19, as well as the use of participant information forms, obtaining informed consent, managing and storing data, and maintaining confidentiality and anonymity.

## 3 Results

### 3.1 The participants—an overview

#### 3.1.1 Demographic characteristics

**Figure 1:**
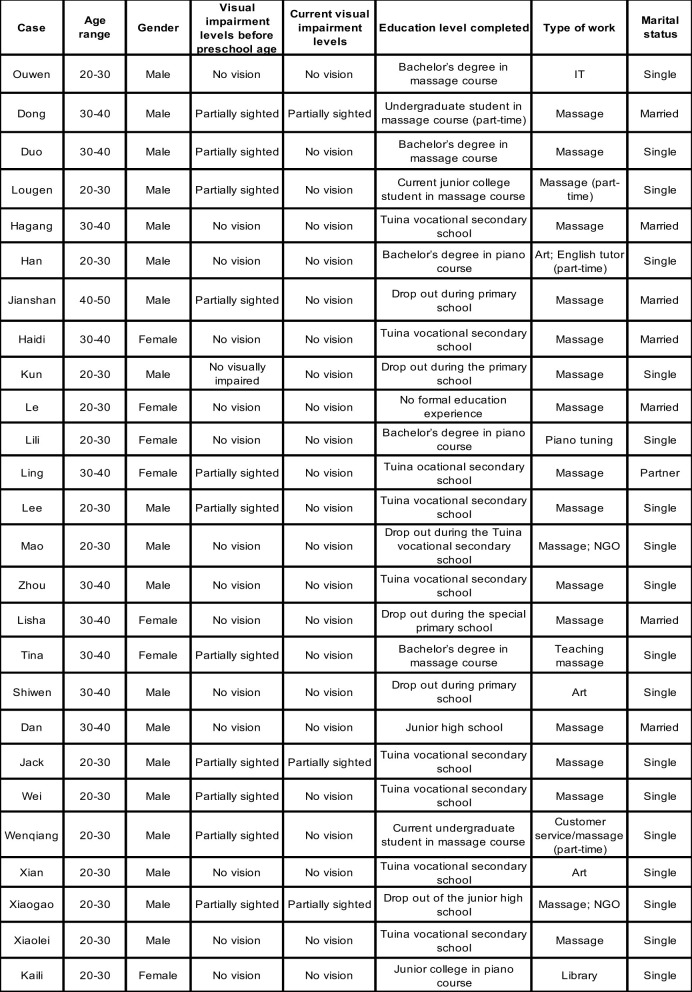
Participants' basic information.

Figure 1 presents an overview of the 26 participants' demographic characteristics, including age, gender, levels of visual impairment (before preschool and at the time of the study), education levels, employment types, and marital status. To ensure anonymity, pseudonyms are used throughout. Participants reported that having no vision or partial sight was a primary reason for rejection from local mainstream preschools or primary schools. For some, progressive vision loss significantly shaped their educational and employment trajectories. Consequently, Figure 1 reports visual impairment levels both before preschool age (typically between ages 3 and 5) and at the time of the study. According to CDPF (2006), visual impairment is classified by hospitals into categories such as “low vision” or “blind.” Only one participant, Kun, reported a specific disability classification (Level 2, severe low vision); others described their impairments using terms such as “born with no vision,” “not sighted,” or “partially sighted” (*banmang* 半盲). To reflect participants' own language, this study uses “no vision” and “partially sighted” in Figure 1.

All participants were born after China's 1978 economic reforms: 13 were born in the 1990s, 10 in the 1980s, and 3 in the early 2000s. They reached school age during a period of expanding services for people with disabilities, following the establishment of the CDPF in 1988 (Feng and Yu, 2017). Despite these shared socio-historical contexts, their educational and employment pathways varied, though certain commonalities emerged, as discussed below.

The sample comprised 19 men and 7 women, a male-to-female ratio of approximately 3:1, primarily from Henan Province in Northern China, with 16 from rural and 10 from urban backgrounds. This gender imbalance contrasts with the Second National Sample Survey of Disabilities in China (The Leading Group for the Second National Sample Survey of People with Disabilities, 2006), which reported a near-equal gender distribution among people with disabilities (51.55% men, 48.45% women). The higher proportion of male participants may reflect lower participation rates of women with visual impairments in education and employment, as noted by Liao and Chen (2018). For instance, Xiaogao shared, “In my primary special school class, there were 14 students, but only two were girls”. Similarly, several male participants noted a scarcity or absence of women with visual impairments in their massage workplaces.

Regarding visual impairment levels, 14 participants had no vision before preschool age, 11 were partially sighted, and one, Kun, had no visual impairment until age 8. At the time of the study, only three participants remained partially sighted, indicating progressive vision loss for most. These impairment levels directly influenced access to mainstream schools and adaptation to learning environments lacking adequate support. For example, Lili, who had no vision, was rejected by a mainstream preschool, stating, “There is no preschool to accept a blind child”. Similarly, Dong, who is partially sighted, withdrew from a mainstream university due to insufficient accommodations, noting, “The university is designed for students without disabilities…. I need to rely on other people's help everywhere.”

Educationally, nine participants completed vocational secondary school with a focus on *tuina* (Chinese medicine massage), nine accessed tertiary education (seven graduates and two current students), and eight discontinued schooling during compulsory education. These varied educational trajectories shaped diverse transitions to employment, particularly into the massage industry.

Regarding marital status, six participants were married, one had a stable partner, three had experienced divorce, and 16 remained single at the time of the interview, yielding a marriage rate of 23.07%. This rate is significantly lower than the national marriage rate for Chinese individuals born in the 1980s and 1990s (56.4%; Global Times, 2017). Notably, single men (84.21%) outnumbered single women (42.86%) in the sample. This disparity suggests that people with visual impairments, particularly men, face challenges in forming long-term relationships. Liu and Xie (2014) and Liu (2017) argue that men with disabilities in rural areas struggle to marry due to unemployment or unstable employment, which they link to traditional expectations of men as family providers. However, this study's participants, most of whom were employed in the massage industry with relatively stable jobs, still reported difficulties finding partners. A unique finding is that all married participants were partnered with individuals who also had visual impairments, a pattern not widely documented in existing research.

#### 3.1.2 Participants' education and work trajectories

**Figure 2:**
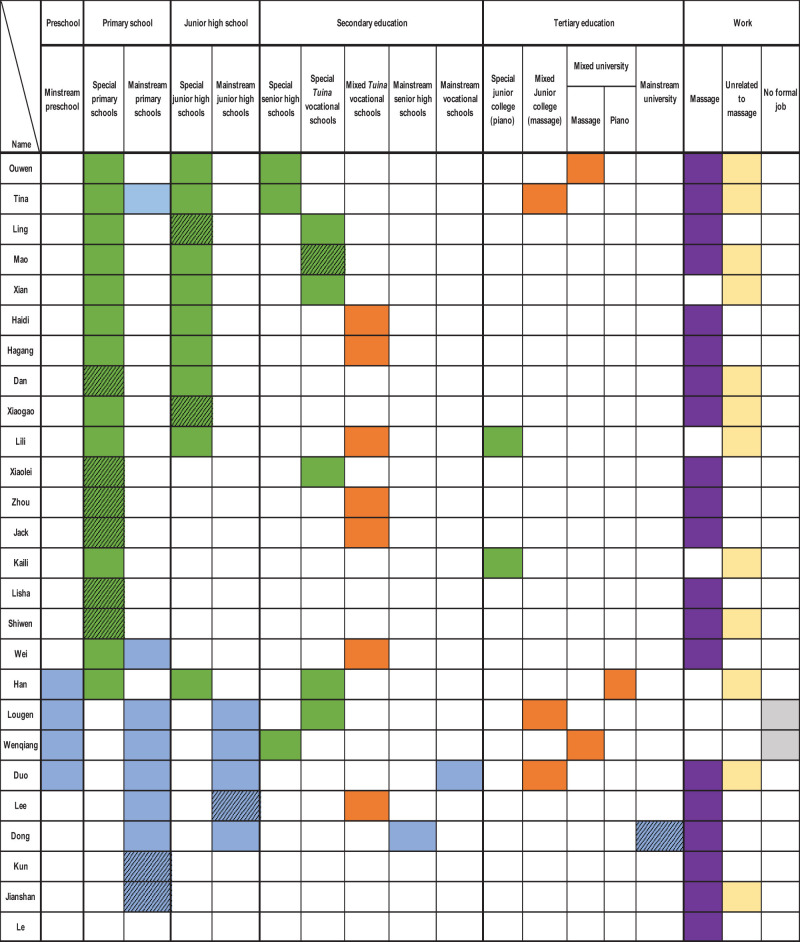
Participants' transitions from education to work.

Given the diverse life courses of the 26 participants, summarising their transitions from education to employment was complex. Figure 2 provides a visual overview of these transitions, illustrating pathways from initial education to employment outcomes. The figure highlights starting points, transitions (including shifts and interruptions), and eventual career destinations, using colour coding to distinguish educational and occupational categories:

**•**
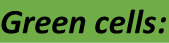
 Special education (e.g., special primary, junior high, senior high, *tuina* vocational schools, or special junior colleges).

**•**
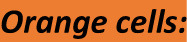
 Mixed education (students with and without disabilities studying together, e.g., mixed tuina vocational schools, junior colleges, or universities).

**•**
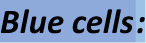
 Mainstream education *(*e.g., mainstream preschools, primary, junior high, senior high, vocational schools, or universities).

**•**
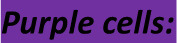
 Employment in the massage industry.

**•**
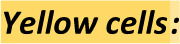
 Work unrelated to massage.

**•**
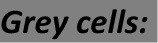
 Participants enrolled in massage training at the tertiary level with no formal employment at the time of the study.

**•**
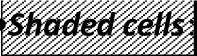
 Dropout from education or training (unshaded cells indicate completion).

Figure 2 maps participants' life trajectories across five educational stages—preschool, primary, junior high, secondary, and tertiary education—aligned with typical ages in China: preschool (ages 3–4), primary (ages 6–7), junior high (ages 12–13), secondary (ages 15–16), and tertiary (ages 18–19). Not all participants experienced every stage. Educational provisions included special, mixed, and mainstream settings. For example, primary and junior high schools were either “special” or “mainstream,” while secondary education included regular curricula (senior high schools) or vocational training (e.g., special or mixed *tuina* vocational schools). Participants frequently switched between provisions, influenced by factors such as school attitudes, parental networks, levels of visual impairment, and the availability of massage training as a primary option.

At the tertiary level, some participants attended special or mixed junior colleges (*dazhuan* 大专) or universities, though course options were limited to *tuina* or piano training. Employment trajectories included three outcomes: massage industry work (predominant), non-massage careers (e.g., piano tuning, customer service), or no formal employment (two participants in tertiary massage training). Shaded cells in Figure 2 indicate dropouts, highlighting incomplete educational stages.

To analyze these complex transitions, two frameworks were applied: entry points into education (three pathways) and career trajectories (typical and atypical pathways). For education, participants followed three pathways: starting in special education (16 participants), mainstream education (9 participants), or no formal education (1 participant). Figure 2 shows that 16 participants began in special primary schools without preschool, nine started in mainstream schools, and one never enrolled in formal education. Notably, 22 participants received massage training and worked in the massage industry, regardless of their starting point.

For career trajectories, one typical and three atypical pathways emerged. The typical pathway, followed by 19 participants (including two current tertiary massage students), involved non-mainstream education (special or mixed) and employment in the massage industry. Atypical pathways included: (a) special education leading to non-massage careers (e.g., Xian, Lili, Kaili, and Han), (b) mainstream education leading to massage work (e.g., Duo, Dong), and (c) no formal education but massage work (e.g., Le). Despite these variations, nearly all participants engaged in massage training, either formally (e.g., vocational schools) or informally (e.g., short-term programs, apprenticeships).

Figure 2 also reveals a low preschool attendance rate, particularly among those starting in special education (only four participants—Lougen, Wenqiang, Duo, and Han—attended preschool). Additionally, 13 participants (50%) dropped out, primarily during compulsory education (primary or junior high), reducing tertiary education attendance. These patterns are further explored in Section 3.2, which details five distinct transition patterns, supported by participant narratives and figures with consistent colour coding.

### 3.2 Five patterns of education-to-work transition

This section examines three patterns of entry into education and two patterns of career transitions, supported by figures using the same colour coding as Figure 2 (green for special education, orange for mixed, blue for mainstream, purple for massage work, yellow for non-massage work, grey for no employment, shaded for dropouts). Text within figures clarifies colour meanings, ensuring accessibility.

#### 3.2.1 Pattern one—participants starting in special education

**Figure 3:**
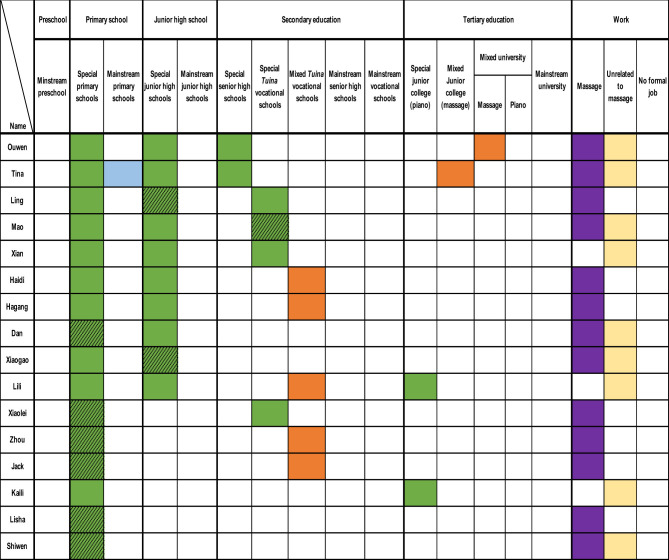
Pattern one—participants starting in special education.

Sixteen participants began their education in special primary schools (green cells, Figure 3), none attending preschool. In China, preschool targets children aged 3–6, typically in standalone kindergartens or pre-primary classes (Su et al., 2021). This absence reflects exclusion from early education compared to peers without disabilities. Entry ages varied, with five participants (Jack, Hagang, Lisha, Zhou, and Ling) starting primary school at or after age 11, later than the typical age of 6–7 (The Standing Committee of the National People's Congress, 1986). After completing or dropping out, 13 participants worked in the massage industry; Xian, Lili, and Kaili pursued creative industries (e.g., music and library work).

Educational transitions varied: nine participants (Ouwen, Tina, Ling, Xian, Haidi, Hagang, Lili, Kaili, and Jack) progressed continuously to work after completing *tuina* vocational or tertiary education. Four (Lisha, Xiaogao, Mao, and Shiwen) dropped out permanently, pursuing informal massage training or non-massage careers (e.g., Shiwen's homeschooling led to creative industries). Three (Xiaolei, Zhou, and Dan) dropped out but returned to special education after working in massage. For example, Liao and Chen (2018) note low admission requirements for *tuina* vocational schools, requiring only a disability certificate, enabling flexible entry.

Two examples of Jack and Xiaolei are given below to show their life storeys (*the italicised text within quotation marks is the direct quotes from participants*), from starting in special education to ending up working in the massage area.

***Jack***, partially sighted, was born in a rural village and raised by his grandparents. He described financial barriers to education: “*Due to my family's financial difficulties at the time, I never considered attending a school for the blind or receiving an education…it simply wasn't something I thought about [he was not able to afford accommodation and meal fees, although the tuition fee was free]. Later, by chance, the local Disabled Persons' Federation [hereafter DPF] somehow obtained my information and contacted me. They provided tuition support, and so, at the age of 12, I began my compulsory education by entering primary school*”. At his province's only special school for people with visual impairments, Jack discovered a talent for piano. However, feeling “too old” at 16, he skipped junior high and enrolled in a technical secondary school for *tuina* training (2 years theory, 1-year internship). After graduating, he worked in the massage industry, briefly playing in a band until it disbanded. Jack attempted to run his own massage parlour but closed it due to low client approval, stating, “*Not many people approved of the massage industry then*”. Currently, he works for employers with visual impairments and plans to reopen a parlour. Motivated by family, he added, “*I hope I can make more money in the massage business, buy a house, and marry a wife; I also hope my grandmother can see all this happen in her lifetime*”.

***Xiaolei***, born with no vision in a poor rural family in the early 2000s, was rejected by a mainstream school. He entered a special school at age 10 but dropped out in Grade 3 at 13 due to family financial pressures: “*My father said the family needed me to provide financial support, so they asked me to learn massage skills instead*”. After a year of private massage training, he worked in the industry for 5 years. At 18, he attended a free *tuina* vocational secondary school funded by the local DPF for systematic training, later continuing in the massage industry. Reflecting on societal attitudes, he noted, “*Blind people do not need compassion, and [people without disabilities] need to help blind people with understanding*”.

Jack and Xiaolei's storeys highlight barriers to education for rural children with visual impairments, particularly when mainstream schools were inaccessible. Both dropped out but pursued massage training—Jack through formal vocational school, Xiaolei via private training—driven by family financial needs and limited career options.

#### 3.2.2 Pattern two—participants starting in mainstream education

**Figure 4:**
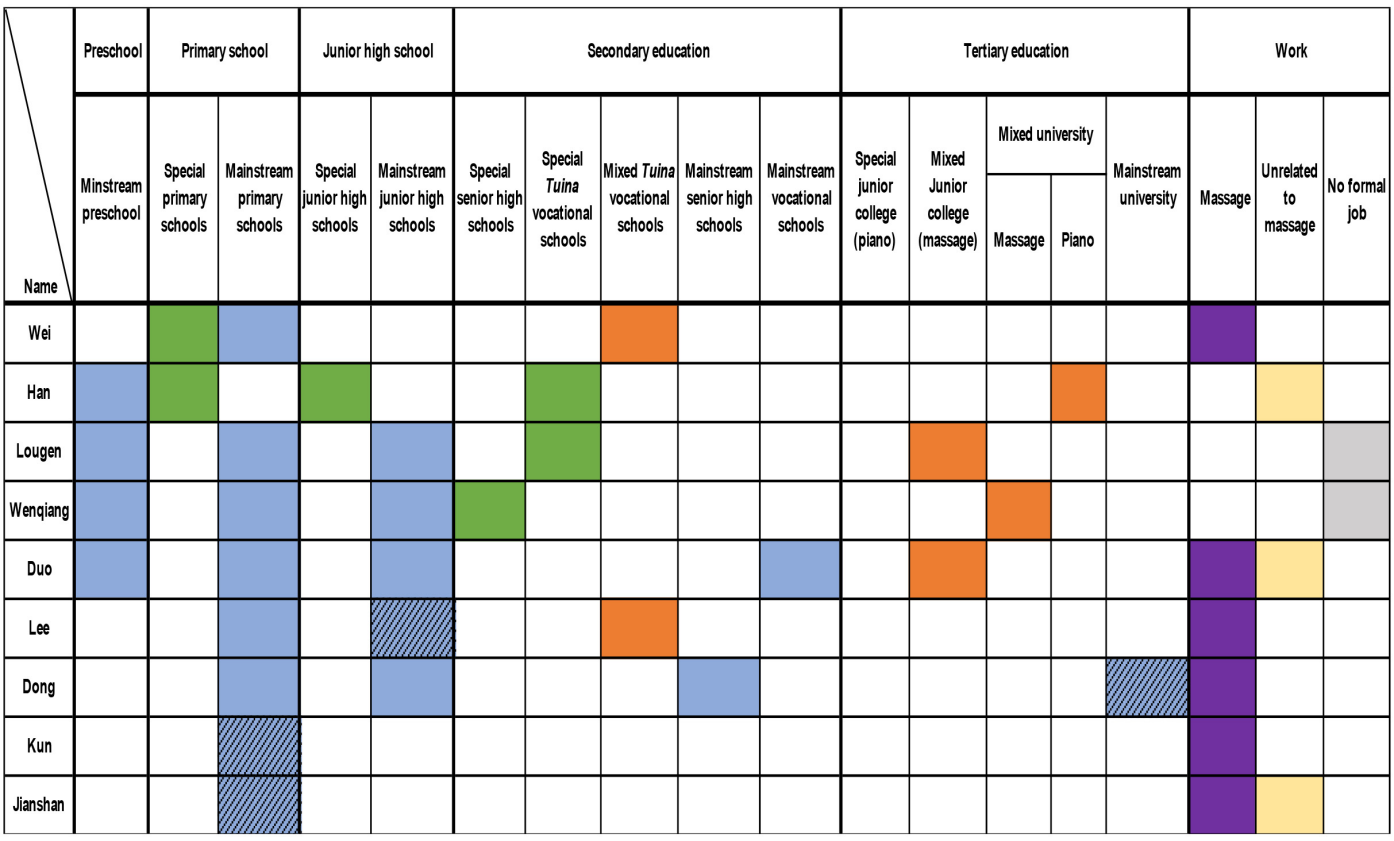
Pattern two—participants starting in mainstream education.

Nine male participants began in mainstream schools (blue cells, Figure 4), eight with partial sight (Wei, Jianshan, Lee, Dong, Lougen, Wenqiang, Duo) or prior vision (Kun), and one with no vision (Han). Four (Han, Lougen, Wenqiang, Duo) attended preschool, aligning with typical ages (3–6). Vision deterioration often marked a turning point (Rutter, 1996), prompting shifts to special/mixed schools or employment changes. Five participants (Wei, Han, Lougen, Wenqiang, Duo) transferred to special/mixed schools due to adaptation challenges or vision loss. For example, Wei transferred to a special primary school in Grade 5 (age 14–15, later than the typical 11–13), later working in massage after *tuina* vocational training. Han, after preschool, attended special schools and studied piano at a mixed university.

Four participants experienced dropouts: Kun and Jianshan left primary school (Grade 1, age 8; Grade 5, age 12), while Lee and Dong returned after dropping out. Kun, after vision loss, learned massage skills at 13, while Jianshan worked in creative industries before entering massage. Lee and Dong resumed education in *tuina* vocational programs after working in unrelated fields.

Below are two exemplar cases of Wei and Jianshan to illustrate how participants starting in mainstream education transitioned from education to work.

***Wei***, not born with a visual impairment but partially sighted after an accident at age 3, attended a mainstream primary school at 8 due to his aunt's teaching role. He described the experience as isolating: “*I felt inferior to others. Maybe I was not willing to socialise with them [classmates without disabilities]. They just laughed at me directly sometimes. Sometimes, I may not say anything, but sometimes, I may get angry and argue with them*”. Transferring to a special school in Grade 5 (age 14–15), he found “*a sense of belonging*”. After completing compulsory education, he attended a mixed *tuina* vocational school and worked in massage parlours, though he disliked the work, stating, “*I would try to find other interests…if I got the chance*”.

***Jianshan***, congenitally blind in one eye, lost all sight after an injury and quit primary school in Grade 5 (age 12). After 3 years at home, he audited his brother's classes, facing bullying: “*They are looking at me as though I am a monkey in a zoo; there were several students who even threw tiny stones at me*”. A teacher recognised his singing talent, leading to a 15-year career with a local Art Troupe for Persons with Disabilities. After a divorce prompted by frequent travel, he trained in massage for 3 months and opened a parlour, later remarrying.

The storeys of Wei and Jianshan highlight bullying and a lack of support in mainstream education for students with visual impairments. They both experienced bullying from some peers without disabilities and found it challenging to continue in the mainstream educational system when there was no assistance. The difference was that Wei had a chance to transfer to special education and then get massage training in formal education. At the same time, Jianshan stopped formally accessing school and worked in the creative industries. However, he finally worked in the massage industry for a steady income/life.

#### 3.2.3 Pattern three—participant with no formal education

**Figure 5:**
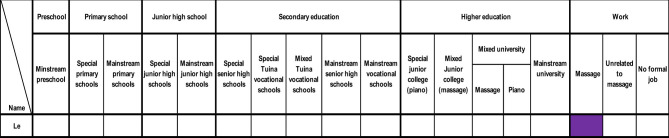
Pattern three—participant with no formal education.

***Le*** was the only participant who never attended any form of formal education in a school setting (Figure 5). She was born in a village and was born blind. Instead, she stayed home until she was 13, when she became an apprentice in a massage parlour recommended by a local villager. Afterwards, she went to different massage parlours to learn massage skills and work in the industry. Finally, she married and opened a massage parlour with her husband. She is now a mother with a preschool child. Although she missed out on education and opportunities, this did not stop her from meeting other important roles in her community, such as employee, wife, and mother.

#### 3.2.4 Pattern four– participants in the typical pathway

**Figure 6:**
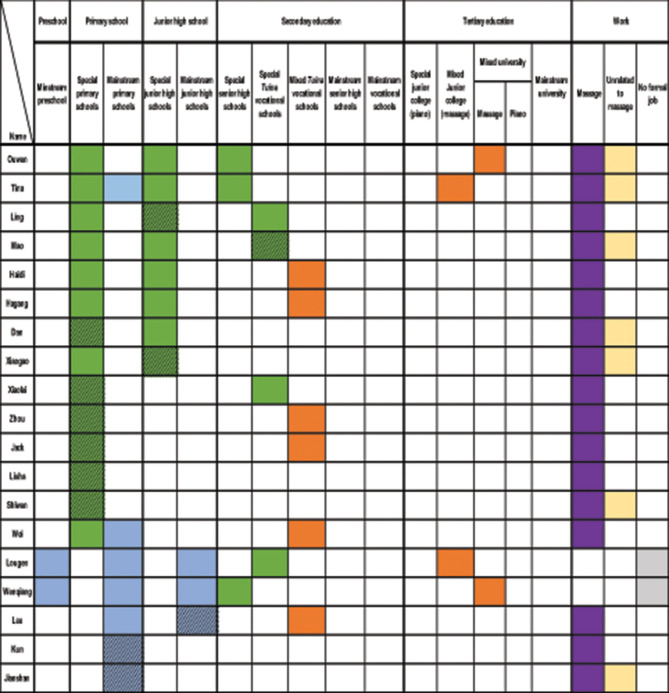
Pattern four—participants in the typical pathway.

Nineteen participants followed the typical pathway (Figure 6), characterised by non-mainstream education (green/orange cells) and massage industry employment (purple cells). Through Figure 6, these participants had a strong connexion with massage, no matter they started special education or mainstream education. There are two broad channels through which they get massage training—through formal education or informal education. Ten accessed formal *tuina* training through vocational or tertiary institutions (e.g., Ouwen and Tina), while others pursued informal training (e.g., Xiaolei's short-term program, Mao's apprenticeship). Under these two channels, participants' educational backgrounds appear unimportant, as they all joined the same industry but in different ways.

Two exemplar cases of **Ouwen** and **Lee** are given below to show how they have connexions with massage training and then worked in this industry:

***Ouwen***, born with no vision in Beijing, attended a special school at age 8, completing compulsory and secondary education. He passed a specialised college entrance exam for people with visual impairments (*dankao danzhao kaoshi*单考单招) and studied *tuina* at a mixed university, including a hospital internship. After a year in a massage parlour, he transitioned to IT, developing games for people with visual impairments, stating, “*I want to work in an area unrelated to massage to eliminate the stereotype that people with visual impairments can only work in the massage area*”.

***Lee***, born in the rural village, partially sighted, attended mainstream schools but dropped out of junior high due to vision deterioration. After working in agriculture and construction, he enrolled in a mixed *tuina* vocational school. He worked in massage parlours for seven years but faced instability during COVID-19. In 2023, he opened his own parlour, expressing concerns about marriage: “*There was a great demand for marriage among people with disabilities, and support was needed*.”

At the end of the interview, Lee expressed his worries about marriage problems. He said there was a great demand for marriage among people with disabilities, and support was needed. He hoped social events aimed at people with disabilities could improve their marriage rate. His parents also worried that if he could not get married and then have children, his later life would be a big problem, as no family members would be around to take care of him.

Ouwen and Lee's storeys showed the channels of access to the massage industry through different educational stages. Ouwen was born in the capital city (Beijing) and, thus, appeared to have more chances to access the formal educational system, including the special senior high school, to learn the regular curricula instead of attending vocational courses like most participants. However, Ouwen still took massage training in higher education and worked in this industry for a while, although he also prepared for other skills and finally worked in the IT area. By contrast, Lee experienced a dropout and then came back to school to attend massage training. As mentioned above, the massage industry is one of the limited choices open to people with visual impairments, whether during their schooling or career pathways, so Lee also chose to get massage training. These contrasting pathways—Ouwen's urban opportunities and Lee's rural constraints—highlight how socioeconomic and geographic factors influence educational and career trajectories for people with visual impairments in China (Hutchison, 2011).

#### 3.2.5 Pattern five—participants in the atypical pathways

**Figure 7:**
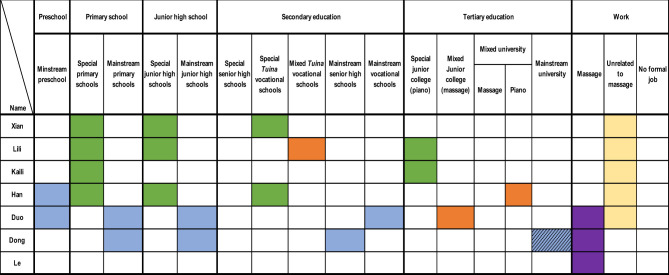
Pattern five—participants in the atypical pathways.

Three atypical pathways emerged for seven participants, as illustrated in Figure 7. The first pathway includes Xian, Lili, Kaili, and Han, who pursued non-massage careers (yellow cells), despite primarily attending non-mainstream schools (green or orange cells) and, for three, engaging in *tuina* training during secondary education. Each developed skills in music (e.g., playing at least one instrument) and other areas, such as English, during their youth. Lili, Kaili, and Han also received home education, practising musical instruments and acquiring literacy skills through parents, tutors, or online resources. These three passed a college admission exam designed for people with visual impairments, enabling them to study piano at a special or mixed institution.

In contrast, the second pathway includes Duo and Dong, who primarily attended mainstream schools (blue cells) but later received *tuina* training (purple cells) through formal education and short-term programs, respectively, leading to massage industry employment. Duo studied in mainstream schools until tertiary education, while Dong attended a mainstream university before dropping out. The third pathway involves Le, who had no formal education but began working as a massage parlour apprentice at age 13, later joining the industry full-time.

Two exemplar cases of **Xian** and **Kaili** are given below to illustrate how they could avoid working in the massage industry despite attending special education:

***Xian*** who lost sight due to hospital malpractice, attended special schools and *tuina* vocational training but pursued music due to family support: “*Doing massage [work] will not be respected by others*”. After exploring *Yijing* 易经 (divination) and online ventures, he joined a Beijing Art Troupe for Persons with Disabilities, later resuming music post-COVID-19. He reflected, “*Many people think that the road of art is not easy to take, but I insist on taking it*”.

***Kaili***, born with no vision, entered a special school at age 9 after her mother's extensive search. She studied at home during secondary education, with private piano and English tutoring. After studying music at a special college, she worked as a Braille librarian, finding it unfulfilling: “*This job was so boring; I was just like a housekeeper.”* There were barely any people with visual impairments coming to borrow Braille books. She complained, “*My duty should serve my clients, but where are my client*s?”. Kaili said her mother always supported her every choice. She said, “*I'm my mother's pride, and my mother's my pride as well*”. She did not like working in the library and still wanted to work in the piano area, but finding that kind of work in her small city was not easy. As a girl with visual impairments, her family also worried about her if she worked in other cities, like how to deal with accommodation and travel issues by herself. Due to this, she has tried part-time jobs online, like writing advertising copy. She also hoped to use her English skills to do some part-time jobs.

Xian and Kaili may have similar backgrounds. They were both born in a city, and each family had a good financial situation, so their family could provide them with private tutoring or extracurricular training to learn skills unrelated to massage. Furthermore, their families seemed to give them a safety net that enabled them to develop skills unrelated to massage without immediately working in the massage industry to support the entire family. This reflects the ways in which families affect the life course of people with disabilities (Aviel et al., 2019).

## 4 Discussions

### 4.1 Comparison of prior research

Through the lens of the life course approach (Elder and Giele, 2009), this study examines the educational and career transitions of 26 people with visual impairments in China, revealing complex pathways shaped by timing, sequence, agency, and social context. Most participants, whether starting in special or mainstream schools, engaged in *tuina* training or employment, reflecting the influence of structural constraints on their life trajectories.

Firstly, the timing of educational transitions significantly shaped participants' life courses. Most were excluded from preschool education, unlike peers without disabilities who typically begin at ages 3–4. Many experienced delayed primary school entry, with five participants (e.g., Jack, Hagang) starting at or after age 11, later than the typical 6–7 (The Standing Committee of the National People's Congress, 1986). One participant, Le, never accessed formal education. This exclusion aligns with gaps in China's Compulsory Education Law (The Standing Committee of the National People's Congress, 1986), which mandates free primary and junior high education (Grades 1–9) but omits preschool provisions. The Law on the Protection of Persons with Disabilities (The Standing Committee of the National People's Congress, 1990) introduced educational rights, yet special schools prioritise compulsory education, limiting preschool access for children with visual impairments. Mainstream preschools often rejected participants. These delayed transitions disrupted the normative timing of educational milestones, impacting learning experiences and career choices, consistent with life course theory's emphasis on timing as a determinant of life outcomes (Elder et al., 2003).

Secondly, the sequence of transitions between education and employment varied, reflecting life course principles of individual agency within structural constraints (Macmillan, 2005). Five participants (Xiaolei, Zhou, Dan, Lee, and Dong) returned to education after periods of employment, illustrating non-linear pathways. For example, Xiaolei dropped out of primary school but later attended a *tuina* vocational school due to family financial pressures. Such sequences highlight how early disruptions influence later life choices, aligning with Macmillan's (2005) focus on the cumulative impact of transition timing.

The dropout rate was high, with 52% (13 of 25 participants, excluding Le) discontinuing education overall and 44% (11 of 25) during compulsory education, compared to a 53.8% completion rate. This is lower than Peng's (2013) reported 72.1% completion rate for children with disabilities in 2011 and far below national mainstream rates (99.27% primary, 97% junior high; Peng, 2013). Recent data report a 95% enrolment rate for children with disabilities from 2017–2022 (The Ministry of Education, 2022), but completion rates remain unclear. Guo (2017) suggests students with disabilities may register without attending, inflating enrolment figures. These disruptions reflect life course disruptions, where structural barriers (e.g., lack of accommodations) alter expected educational trajectories (Elder, 1998).

As far as the curricula they studied are concerned, the participants here mainly followed the regular curricula (i.e., Chinese, Maths, English, History, etc.) as part of the 9 years of compulsory education. The vocational curricula (related to *tuina*) participants studied were primarily in secondary and tertiary education. Half the participants (13) followed vocational curricula to take massage courses in the special/mixed vocational senior high school; nine participants got massage training or learned piano in their tertiary education. This supports the findings of Kritzer (2011) that students with visual impairments in China are generally provided with massage or piano courses in tertiary education. In addition, the rate of higher education enrolment is just 34.6% (nine out of 26), which is lower than the national average of 57.8%, reported by People's Daily (*Renmin Ribao* 人民日报) (2023). The newspaper report also claims that “higher education has entered the stage of popularisation”, which may not apply to people with disabilities. From a life course perspective, these limited educational options reflect the principle of linked lives, where social structures, particularly China's educational system, constrain individual life choices (Elder et al., 2003). The school system's focus on *tuina* and piano training, driven by societal assumptions about suitable careers for people with visual impairments, funnels participants into predetermined vocational paths, limiting their ability to pursue diverse professions.

The principle of linked lives further illuminates how family influences, intertwined with urban vs. rural contexts, shape participants' opportunities for non-massage skills and careers, despite shared social barriers (e.g., mainstream school rejection and *tuina* training predominance). Urban participants, like Xian and Kaili, benefited from family resources that facilitated access to alternative skills (e.g., *Yijing*, music). Xian's parents supported his music training, enabling his transition to the creative industry, while Kaili's mother arranged private piano and English tutoring. These urban families, with greater access to educational networks and financial resources, expanded participants' options beyond *tuina*. Conversely, rural participants, like Xiaolei and Jack, faced family pressures to prioritise financial stability, often pushing them towards *tuina* training due to limited local opportunities. Xiaolei's family, for instance, prioritised income over education, leading to his early dropout. Despite these differences, all participants faced systemic constraints—illustrating how linked lives connect individual trajectories to family and societal influences (Elder et al., 2003). Urban families' support enabled some to navigate these barriers towards non-massage careers, while rural participants were more tightly bound to *tuina* paths, highlighting the interplay of agency and structural constraints (Feng and Yu, 2017).

Some findings have not been discussed in the prior research yet. Regarding the channels for obtaining massage training, in addition to receiving formal education in massage training, the participants accessed the massage training through informal education, including taking a private short-term massage training project and being an apprentice in the massage workplace. However, all participants who started in mainstream education studies worked in the massage industry; this means that those mainstream educational settings can offer them a limited range of career choices. Additionally, no matter how their life experience and transitions differed, almost every participant has learning/working experience in the massage area. Most (17 out of 26) had massage work as their main career. A few ventured into other regions due to their additional skills, such as IT and music. For example, Ouwen emphasised agency in challenging these constraints—working in an area unrelated to massage to eliminate the stereotype that people with visual impairments can only work in the massage area. This reflects life course agency, where individuals navigate structural barriers to redefine their trajectories (Feng and Yu, 2017). Some even experimented with different jobs before returning to the massage industry, a testament to the industry's appeal and challenges in other fields. This result also showed that it is difficult for people with visual impairments to break the culture of vocational stereotypes, and they usually do not have many career development choices.

### 4.2 Theoretical and practical implications

A key theoretical contribution of this research is the application of the life course approach to Chinese disability studies. The current life course studies on disability studies mainly focus on Western culture, but it has been underutilised in disability research within China. This study, therefore, provides a novel contribution by using this framework to explore the lived experiences of people with visual impairments in Chinese contexts. The findings highlight how structural constraints, such as limited preschool access and vocational stereotypes, shape life trajectories, extending Elder's (1998) framework to a non-Western context.

On a practical level, the research highlights how individuals with visual impairments navigate transitions into education and employment. It reveals that participation in mainstream education does not necessarily divert them from employment in the massage industry. In fact, receiving massage training remains a near-inevitable path for people with visual impairments in China, regardless of whether the decision is voluntary or externally influenced. This suggests a need for inclusive preschool programs, accommodations in mainstream schools, and expanded tertiary options to enhance agency and challenge vocational stereotypes.

### 4.3 Limitations and future directions

This study has aimed to explore the transition experienced by people with visual impairments as they transition into education and work. 26 participants were recruited (including males and females) with work experience. However, the male-to-female ratio of the participants was three to one. According to the participants, more males of all people with visual impairments enrol in all school levels (primary, secondary, and tertiary education). It is quite possible that, like participant Le in the study, many females with visual impairments never attend formal education settings. The life storeys of these “invisible” females still need to be further explored.

## Data Availability

The raw data supporting the conclusions of this article will be made available by the authors, without undue reservation.
